# Suprathel Versus Hypafix in the Management of Split-Thickness Donor Site Wounds in the Elderly: A Randomised Controlled Trial

**DOI:** 10.3390/ebj5040031

**Published:** 2024-10-17

**Authors:** David Cussons, Justine Sullivan, Quentin Frew, David Barnes

**Affiliations:** St. Andrew’s Centre for Plastic Surgery and Burns, Mid and South Essex NHS Foundation Trust, Chelmsford CM1 7E, UK

**Keywords:** epidermal skin substitute, Suprathel, split-thickness skin graft, wound healing

## Abstract

(1) Background: Effective wound management aims for expedited healing, improved functional and scar outcomes, and reduced complications including infection. Delayed wound healing remains a prevalent problem in the elderly. Suprathel is a synthetic absorbable skin substitute and an attractive option in partial thickness wounds. The objective of this randomised controlled study was to assess the effect of skin substitute dressings on elderly split-skin graft (STSG) donor sites, evaluating time to heal, pain, itch and scar outcome. (2) Methods: 40 patients over 65 undergoing split-thickness skin grafting for non-melanoma skin cancer excision were randomised to STSG donor site dressings with either Suprathel or Hypafix. Patients were followed up weekly until healed and at 13 weeks post-procedure. (3) Results: There was no significant difference in time to healing, pain, itch, or scar outcome at 13 weeks between the two groups. The mean time to healing was 31.7 days for the skin substitute group and 27.3 days for the adhesive tape control group (*p* = 0.182). (4) Conclusions: Both dressings are appropriate for STSG donor sites. Hypafix remains a cost-effective dressing of choice for donor sites. Benefits demonstrated in other studies using skin substitutes have not translated into the elderly population. There remains scope in developing dressings that reduce elderly donor site morbidity.

## 1. Introduction

Reconstruction with split-thickness skin graft (STSG) remains the optimum surgical strategy for achieving skin coverage in large defects which have a vascular wound bed. Skin grafts are used extensively in a variety of disciplines including burns and complex wound reconstruction after trauma or oncological excision; however, STSGs are associated with significant donor site morbidity, including pain and itch as the wound heals, infection, and delayed wound healing. This can lead to increased dressing burden, altered pigmentation, and scarring in the long term [1,2,3]. 

The wound healing process can be divided into haemostasis, inflammation, proliferation, and tissue remodelling. STSG donor sites are partial thickness wounds and are able to recruit keratinocytes from adnexal structures and the wound edge and thereby heal faster than full thickness counterparts. With dermal preservation, the extant basement membrane provides a vascularised bed for this re-epithelialisation to occur. In the elderly population especially, biochemical and immunological changes in the inflammatory stage of this process including increased concentrations of inflammatory mediators and age-related changes in immune cell recruitment can have profound effects on wound healing and lead to substantial delay [4,5].

Current management of STSG donor sites is dressing based, with ideal dressings possessing a number of important characteristics. Traditional donor site dressings are left on to the point of re-epithelialisation and must be wear resistant as well as absorbent enough to manage wound exudate; this is a particular concern if the surrounding tissues have been infiltrated intra-operatively to improve graft harvest. The ideal dressing is also non-adherent, haemostatic, and minimises pain and trauma to the patient and the healing area. Finally, in a responsible environment, cost-effectiveness also plays an important part in appropriate dressing selection.

Hypafix is an adhesive tape dressing containing a water-insoluble adhesive and has been a mainstay of donor site wound care for more than 35 years [6,7]. The water-insoluble adhesive allows for steam sterilisation of the dressing and therefore a sterile contact layer against the donor site wound. Furthermore, adhesive tape also permits exudate to pass through to a secondary dressing layer, and whilst the outer layers can be changed, the base layer is left in contact with the wound bed until fully healed. Adhesive tape can, however, be challenging to apply, with creases in the material leading to exudate run off and premature dressing changes. There has also been considerable debate about patient comfort in the context of adhesive tape, with competing arguments over the pain experienced post-operatively with the use of adhesive dressings [8,9].

Suprathel is a skin substitute dressing based on a series of synthetic co-polymers [10]. It is described as a permanent absorbable dressing that can be left on from initial definitive management until the wound is healed, and it offers the promise of reduced pain and post-surgical complications. Whilst Suprathel has been demonstrated to be benefit in partial thickness burns, it has been used on other partial thickness wounds such as skin graft donor sites and following toxic epidermal necrolysis [11,12,13,14,15]. Although various studies have demonstrated reduced pain, improved scar outcomes or reduced time to healing, none of these outcomes have been consistently demonstrated in the current literature. The heterogeneity of these wounds complicates efforts to synthesise competing results from multiple studies [16]. The majority of studies have also been in the paediatric or adult burn populations, and these wounds often display heterogeneity even within individual patients, where wound depth and therefore expected time to heal changes across the surface of the wound bed. The current literature for elderly patients is also lacking and given the discussed age-related differences in wound healing studies in adult populations may not necessarily be applicable to this patient group.

Management of the partial thickness wound aims to minimise time to healing and therefore long-term morbidity associated with symptomatic or disfiguring scarring [17,18,19]. This is of particular concern in the elderly, where changes in the microscopic inflammatory environment, patient co-morbidities, and functional status may contribute to slower wound healing and therefore poorer post-surgical outcomes [20,21]. Our objective was therefore to investigate whether skin substitute dressings would confer this advantage on elderly STSG donor site wounds.

## 2. Materials and Methods

The trial was performed as a prospective, randomised, unblinded, non-inferiority investigation to study the impact of skin substitute dressing choice on slow to heal uniform depth dermal injuries, represented by STSG donor sites in patients over 65 years old.

All participants were patients undergoing non-melanoma skin cancer surgery with proposed split-skin graft reconstruction at a tertiary burns and plastic surgery centre. Patients confirmed participation in the study by signing the informed consent form.

Inclusion criteria for patients were patients 65 to 100 years of age, undergoing less than 2% total body surface area (TBSA) split-skin grafting, and able to provide informed consent to the study.

Exclusion criteria were patients outside the above age range or any allergy to either of the dressings used in the study. Patients with any medical condition such as immunosuppression, poorly controlled diabetes, or peripheral vascular disease predisposing altered wound healing were also excluded from the study.

The treatment arm consisted of a donor site dressing with Suprathel, paraffin impregnated gauze, dressing gauze, and sterile Hypafix. The control arm consisted of a donor site dressing with sterile Hypafix, dressing gauze, and sterile Hypafix, representing the current standard of care.

A total of 40 envelopes were prepared for randomisation, each was filled with one of two alternative texts to randomly allocate patients to either the skin substitute or adhesive tape study arm, shuffled and sequentially numbered. Randomisation occurred on the day of surgery prior to the surgical procedure. Neither the patient nor the surgical team were blinded as to the dressing used.

**Figure 1:**
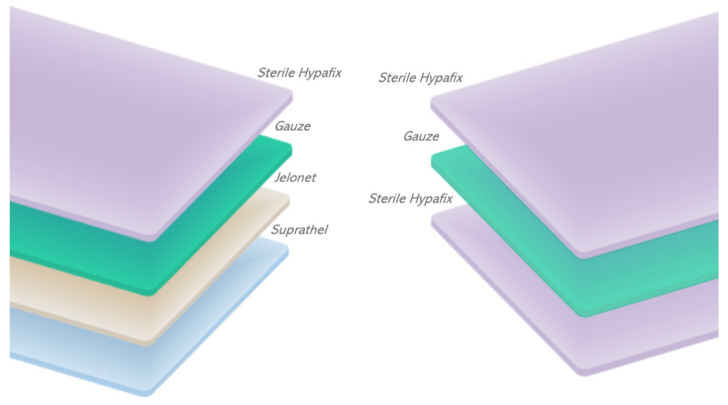
Illustration of the dressing layers applied to STSG donor sites in each treatment group.

STSGs were harvested from patient’s proximal anterolateral thighs at 0.20 mm (8 thousandths of an inch) using a Zimmer Air dermatome and paraffin lubricant. Donor sites were dressed by surgical staff familiar with both dressings. The base dressing layer of either sterile adhesive tape or skin substitute was cut to fit at least 2.5 cm beyond the edges of the donor wound, overlaid with paraffin impregnated gauze in the case of the skin substitute dressing, then with several layers of dressing gauze and a layer of sterile adhesive tape to secure the dressing in each case. Skin substitute sheets are available in 5 cm by 5 cm, 9 cm by 10cm and 18 cm by 10 cm sheets, and the smallest sheet able to provide wound coverage was used in each instance. Adhesive tape sheets are available in 40 cm by 20 cm sheets, and in each case, a single sheet was used to secure the dressing. Image representation of the dressings used is supplied in Figure 1.

Patients were followed up weekly from week two in a specialist plastic surgery dressing clinic until the point of healing, defined as >95% of donor site healed. Any adhered basement layer was left in place over the donor wound, with loose free edges trimmed back. Any area where the original dressing had separated from unhealed wound was trimmed away and replaced with a simple dressing including a silicon based non-adherent base layer.

At each weekly dressing appointment, photographs were taken of the donor site. Patients reported pain and itch on a 10-point scale, as well as patient and clinician reported outcome measures in the form of POSAS v2 from the first and each subsequent appointment at which healed areas were available for evaluation [22].

Time to healing was defined in days from surgery to the first appointment at which the donor wound was >95% healed.

Participants attended a final appointment at 13 weeks following surgery, undergoing photography, POSAS evaluation, and assessment of pain and itch. POSAS records patient and observer reported data points about pain, itch, as well as scar colour, thickness, surface area, pliability, and relief. A score of 0 represents normal, with 10 being the highest possible morbidity.

Cost analysis was performed based on list prices per box of dressings from each dressing supplier excluding VAT, cross-referenced with previous cost estimates where available [12,23].

Study group sample sizes of 20 participants each were derived in consultation with an independent statistician. Calculations were based on a non-inferiority test with an estimated healing time of 14.3 days and a standard deviation of ±2.9 days for both groups, aiming to capture a significant difference in time to healing of ±2 days, giving a power value of 0.9 with 20 participants a group or 0.8 with 14 participants per group, accounting for attrition.

## 3. Results

A total of 40 patients were randomised. Participants had an average age of 79.8 years, split male to female 22:18. The average donor site surface area was 37.62 cm^2^ across both groups, with a slightly smaller average donor site in the adhesive tape group (32.01 cm^2^) than the skin substitute group (42.92 cm^2^). Patient demographics by study arm are displayed in Table 1. Distribution populations for both sample sets were similar when analysed with a two-sample Kolmogorov–Smirnov test (age, *p* = 0.560, surface area, *p* = 0.510) and the difference in surface area between the two groups did not represent a significant sampling difference between the two study arms.

**Figure 2:**
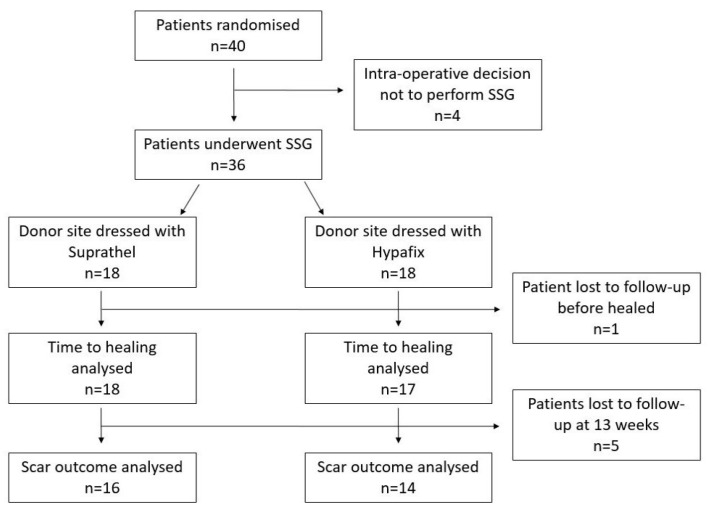
Flow diagram of participant randomisation and follow-up through the study.

All participants were day case procedures for excision of non-melanoma skin cancer. All patients were Fitzpatrick skin type I–III. Four patients were removed from the study after the intra-operative decision not to perform a skin graft post-consent and randomisation, with lesions closed directly in two cases and in two cases with a local flap. One patient was lost for immediate follow-up at week 3, with five further patients declining to attend the week 13 follow-up. Patients who declined to attend follow-up cited unwillingness to return to a tertiary centre in all cases, with particular regard to travel times and worsening medical conditions unrelated to skin cancer surgery. In total, 35 patients were followed up until healed, with 30 patients completing a final week 13 follow-up as demonstrated in Figure 2.

Parametric data were analysed with a two-sample *t*-test. Time to healing was analysed with a Wilcoxon signed rank test. There was no statistical difference in mean time to healing: 31.7 days for the skin substitute group and 27.3 days for the adhesive tape control group, with a mean difference of +4.4 days to heal with the skin substitute. A total of 10 patients (55.6%) took longer than 3 weeks to heal in the skin substitute group against 9 patients (50%) in the adhesive tape control group. There were no donor-site related post-operative complications or adverse events in either group.

**Figure 3:**
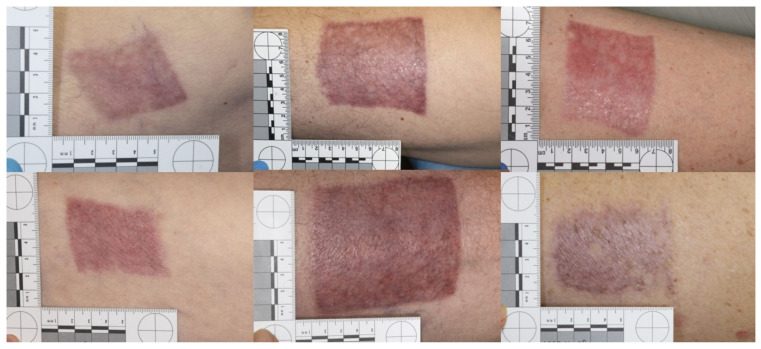
Consecutive patient examples for donor site scar outcome at 13 weeks post-graft harvest. The top row photographs are patients treated with skin substitute dressings. The bottom row consists of patients treated with sterile adhesive tape.

**Figure 4:**
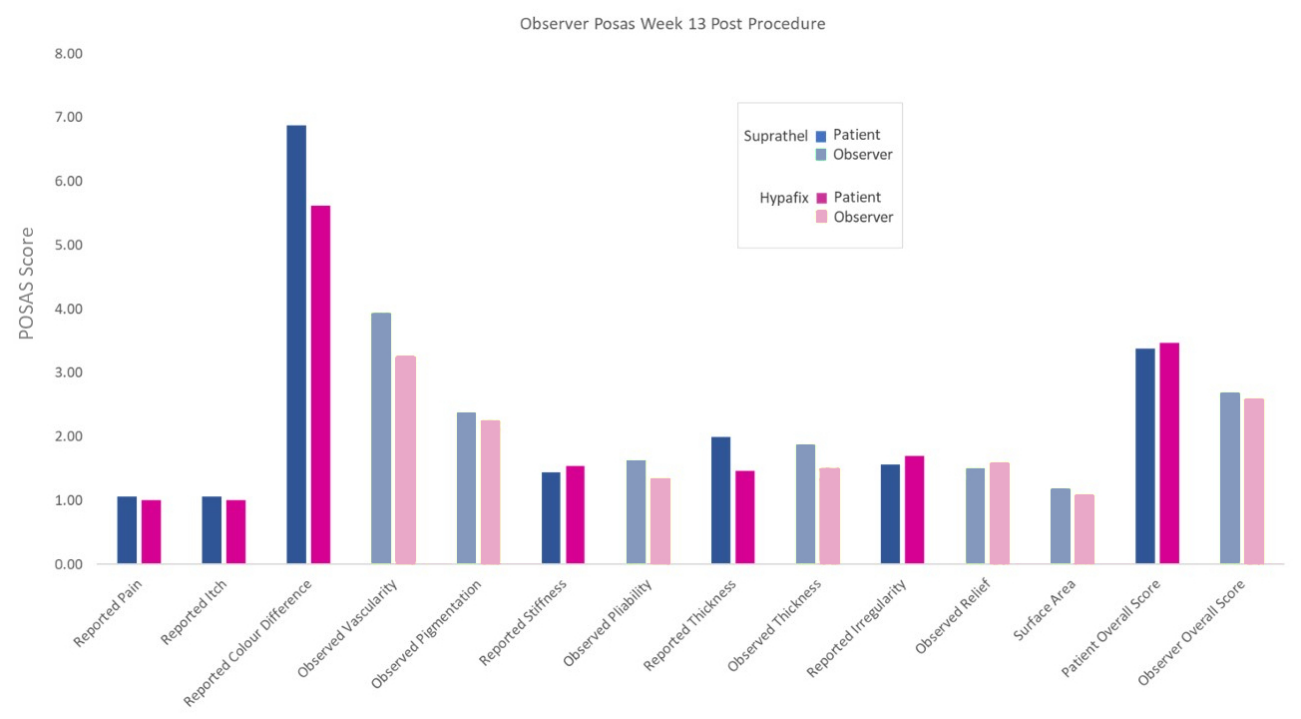
Means for both patient and observer reported POSAS in each study arm at 13-GBP w-up post-procedure, grouped by characteristic.

POSAS results at final follow-up 13 weeks post-procedure were also not significantly different between the two groups, with overall observer scores of 2.69 for skin substitute and 2.58 for adhesive tape control and overall patient scores of 3.38 for skin substitute and 3.46 for adhesive tape control. Photography at week 13 for consecutive patients in both the skin substitute and adhesive tape groups is displayed in Figure 3 for the purposes of visualisation of the POSAS scores. Full breakdown for each section of the POSAS score at 13 weeks post-procedure are presented in Figure 4 for patient and observer characteristics.

Patients reported the biggest deviation from norm for colour at 13 weeks post-procedure, with scores of 6.88 for the skin substitute and 5.62 for adhesive tape. Pliability was close to normal in both cases, both for patients (1.44 for the skin substitute vs. 1.54 for adhesive tape) and observers (1.63 for the skin substitute compared to 1.33 for adhesive tape). Relief reported by patients was 2.00 for the skin substitute against 1.46 for adhesive tape, reported by observers as 1.50 for the skin substitute and 1.58 for adhesive tape. Observers also reported comparable pigmentation (2.38 for the skin substitute against 2.25 for adhesive tape) and vascularity (3.94 for the skin substitute against 3.25 for adhesive tape). In all cases, results were comparable between groups and not statistically significant and are displayed in Table 2.

Based on the dressings required to cover each donor site, the average cost of the skin substitute dressings was GBP 57.99 vs. GBP 3.50 for adhesive tape dressings, for a cost differential of GBP 54.49. The average list price for skin substitute dressings used in the study was GBP 0.57/cm^2^, whereas the average list price for adhesive tape dressings was GBP 0.002/cm^2^. Accounting for overlap of dressings onto normal skin and the average surface area of each wound as well as wastage for oversized dressings, actual price for skin substitute dressings per square centimetre of wound covered was GBP 1.54/cm^2^. Given the larger adhesive tape sheets available for this study, there was a larger increase in adhesive tape waste for an actual price per square centimetre covered of GBP 0.08/cm^2^.

## 4. Discussion

This study has demonstrated equivalence in time to healing between Suprathel and Hypafix dressings following split-skin graft harvest. There was also parity in the secondary outcomes of pain or itch at one week post-dressing or scar outcome 13 weeks post-procedure.

The study had a randomised, prospective, controlled, and un-blinded design. It would have been desirable to conceal the basement dressing used from both the patient and the clinical team but given the difference in appearance of both dressings, this was not possible. In this context, therefore, a non-inferiorty design allowed for the assessment of both healing time and patient-reported outcome measures against current practice. The potential bias introduced by this is limited by the fixed depth at which donor skin was harvested as well as an objective primary outcome measure of time to healing for the donor site wound. It is possible that patients were biased in their reporting of pain and itch post-procedure based on their knowledge of which dressing had been applied, and there is equally a chance of patient or clinician bias in the reporting of the POSAS questionnaire at the final visit.

Whilst the use of donor sites in this methodology has the advantage of setting all wounds at a fixed depth and making them more directly comparable, it does limit the assessment of wounds in this study to superficial partial wounds. Suprathel has also been demonstrated to be of use in deep partial thickness wounds and this is not assessed in this study [24]. There is, however, a substantial body of evidence examining superficial partial burns or donor site dressing with Suprathel, although not as randomised controlled trials with a direct comparison, and this paper provides greater context for that work and underlines the particular benefit of this dressing choice in deep dermal injuries [9,18,25]. The use of patients undergoing excision of non-melanoma skin cancer in this study allowed for otherwise limited selection bias due to the large available population, and obviates unfavourable and heterogeneous patient factors more prevalent in the burn and trauma populations.

Objective measures of scar outcome such as colourimetry, pliability, and ultrasound obviate the issue of confirmation bias but do not capture the patient perspective [25]. In this setting, POSAS as a patient recorded outcome measure has been shown to provide adequate information at 13 weeks post-procedure to predict long term scar outcome in partial thickness wounds [26]. Inter-rater variability is, however, a documented problem with subjective scar assessment, and it was for this reason that the POSAS assessment was performed by staff trained to use POSAS and the same assessor was maintained for all patient follow-ups with photographic documentation [11].

Six patients were lost for follow-up over the course of the study, with the main reason cited by patients being the challenge in returning considerable distances for follow-up to a tertiary plastic surgery centre. These patients were split evenly between the two groups, limiting any potential biasing of the week 13 POSAS. Only one of six patients failed to continue follow-up before being healed due to an unrelated decline in health, and results from the other five patients are included within the data on time to healing as the primary outcome measure. The attrition rate experienced here is a limitation of the study but comparable with other populations investigating this age group. Furthermore, the study remains adequately powered at a value of 0.8. The study participants who declined to attend at week 13 follow-up confirmed this during telephone interviews, and all denied any complaint regarding their long-term scar outcome.

There is a substantial body of evidence documenting delays in wound healing associated with age. Normal wound healing is altered, with a prolonged inflammatory phase and increased concentrations of inflammatory mediators including TNF-α, TGF-β, and PDGF [27,28]. This is paired with reduced rates of macrophage adhesion and consequently reduced rates of granulation and angiogenesis [5]. At the same time, flattening of the dermis and thinning of the epidermal layers may also result in set-depth wounds created by a mechanical dermatome leaving comparatively fewer reservoirs within the donor site from which to re-epithelialise the resultant wound bed [5,29]. Elderly patients are also more likely to have co-morbidities that may influence wound healing such as micro-vascular disease, diabetes and cardiac disorders requiring anti-coagulation [4]. Finally, the hormonal environment changes with age and decreases in production of sex hormones in the elderly have been demonstrated to contribute to prolonged wound healing [30]. Inadequate nutrition can also contribute to poor wound healing, and this was not controlled for, as participants all ate their regular diets at home; however, no participants had a documented history of malnutrition. Females over 65 are at particular risk of deficiencies involving folate in the United Kingdom; however, any such risk is likely to be mitigated by the randomised nature of the trial [31].

Risk of hypertrophic and problematic scarring has been demonstrated to increase with prolonged time to healing, with increased markedly hypertrophic scarring occurring beyond the 3 week mark [32]. This is of particular concern over the time frames of this study, with the average time to healing across both groups being 30 days. It is therefore plausible that in this unfavourable host environment, it becomes more important to optimise controllable factors in wound healing including choice of dressing. Application of suitable dressings may have an outsized impact on wound healing in comparison to moderation of the young healthy donor site. Indeed, time to healing was significantly longer in our population than means published in other studies with younger populations for both study groups, but the finding of no change in time to healing between dressing types broadly consistent with the current literature [9,12,13,33,34,35,36]. Importantly, the rate of hypertrophic scarring was low in this population, with thickness recorded at below two for both reporter and observer thickness. This highlights a particular research desert with present studies investigating prolonged wound healing looking at young adult or paediatric populations [32,37,38].

Patients were generally satisfied with the long-term appearance of the donor site scar, reflected in the low patient-reported POSAS categories both by characteristic and overall, with the notable exception of the colour of the scar, regardless of the dressing used. This finding corroborates evidence in the adult and paediatric burn population [9,24,32,35,36]. A minority of studies performed on STSG donor sites have reported improved scarring against POSAS and VSS tools, at odds with the results displayed here [13,33]. Although POSAS is a sensitive tool, it remains subjective, and low scores may reflect the fact that following skin cancer excision patients are generally appreciative of surgery and willing to tolerate some level of cosmetic defect. This highlights the importance of management approaches that consider individual patient priorities and experience. It is also possible for patients to learn POSAS over the course of repeat assessments and this could also bias results, but with weekly dressing changes, the number of opportunities for each patient to complete POSAS self-assessment was limited and likely a relatively small component of subjective variation in the context of lived scar experience.

There was also equivalence between study groups in pain and itch scores. Other studies have identified reduced pain as a benefit of Suprathel dressings, especially in the paediatric burn population [12,13,14,33], but these findings do not seem to translate into benefits in elderly donor site wounds despite donor site pain in the early stages post-procedure being a common issue for these patients [2,3]. Whilst initial scores at 1-week post-surgery were elevated in both groups, these tailed off quickly on subsequent appointments. The low average scores for both groups reflect the fact that both dressing types were well tolerated, with minimal functional and psychological impact. This study was performed in relatively small donor sites (<1% TBSA) and puts a lower bound on the donor area for which dressings that minimise pain are important. It is possible that patients with more extensive donors derive greater benefit from dressings such as Suprathel. Finally, the first data point recorded in this study was at 1-week post-procedure, and more granular patient reporting of pain scores in the first days following operation may delineate better differences between dressings, although the pain reported here shows that any benefit is likely to be nullified by 1 week. There were also no post-operative infections in the observed population, which can be a key cause of delayed healing and hypertrophic scarring, recognized as contributing to increased morbidity in other studies [14,34].

Whilst both dressings are relatively easy to apply, creasing and bleeding under dressings when applying adhesive tape may lead to areas of the wound not in contact with dressings, a problem not present when applying Suprathel. Suprathel must be covered with a layer of Jelonet, and therefore requires nursing staff to be trained to treat both layers as the primary dressing with no separation. The intrinsic adherent properties of Suprathel may provide additional benefit in larger donor sites, where creases in adhesive tape may lead to wound exudate run off and soaked dressings. However, Suprathel may fail to adhere to overly deep wounds, and where wounds deepen or slough away any advantages of Suprathel may be negated [34]. Suprathel has also been shown to be an effective dressing in superficial partial thickness burns, and this may confer additional ease of dressing in patients with concomitant donor site and burn wounds on the lower limbs [12,14]. Suprathel has also been demonstrated to be of use in deep partial thickness wounds, possibly conferring additional advantages in the burns patient where it is able to dress both mixed depth burn wounds and donor sites.

At time of writing, Suprathel is available at a cost of approximately GBP 0.50/cm^2^ of dressing. Whilst this is considerably more expensive per area covered than Hypafix by orders of magnitude, the price differentials in this study remain relatively modest in absolute terms. There was a substantial increase in cost in this study due to the small wound size and available size of dressings which may translate to a lower cost differential in bigger wounds. In a resource constricted environment, the increased cost of skin substitute over adhesive tape is a consideration when choosing donor site dressing given the equivalency between the two dressing choices, especially given the characteristics of sterile adhesive tape that make it an attractive donor site dressing: It remains relatively easy to apply, results in an acceptable amount of pain post-operatively, can be removed with appropriate solvents such as plant based oil where necessary, and although it has no specific haemostatic effects, it is capable of managing bleeding and exudate when paired with an absorbent layer Nevertheless, where patient factors and the particular properties of Suprathel we have discussed are relevant, the moderate added cost of the single dressing may be justified if it reduces the cost associated with other interventions [12]. Due to the identical follow-up period of both patient groups in this study design, cost differentials owing to fewer appointments and changes in nursing time were not captured here, and the idealised wound bed created by STSG donor sites here may obscure benefits associated with fewer dressing changes in more complex or hard to heal wounds.

## 5. Conclusions

Suprathel and Hypafix are both appropriate dressings for split-thickness donor sites. Adhesive tape remains a cost-effective dressing of choice for managing donor sites. Time to healing and pain benefits suggested by other studies in partial thickness injuries using skin substitute have not translated into a clear demonstration of superiority over standard of care in the relatively small donor sites and elderly population used in this study. There remains scope for development of dressings that reduce elderly wound associated morbidity.

Even with clearly defined treatment goals and a range of suitable products available, there is still no definitive standard of care in the management of donor site wounds. This study demonstrates that even within a homogeneous population of wounds, patient factors such as age generate distinct sub-populations with altered wound healing, and results obtained in adult and paediatric cohorts are not necessarily applicable. A larger multi-arm study sufficiently powered to be able to differentiate these factors within a larger population would demonstrate the specific impacts of different dressing considerations.

## Figures and Tables

**Table 1:** Demographic data for both patient groups, K-S Distribution function refers to Kolmogorov–Smirnov test for equality of distribution.

	Skin Substitute	Adhesive Tape	K-S Distribution Function	*p*-Value
Age	80.9	78.8	0.452	0.397
Male:Female Ratio	12:8	10:10		
Average Donor Surface Area	42.92	32.01	0.400	0.204

**Table 2:** Outcome measures for both patient groups. Statistical tests are t-tests for parametric data. Days to healing measured with Wilcoxon signed-rank test.

	Skin Substitute	Adhesive Tape	*p*-Value
Patients followed until healed	18	17	
Patients followed up to week 13	16	14	
Days to heal	31.7	27.7	0.182
Donors longer than 3 weeks to heal	10 (55.6%)	9 (50%)	
Itch at week 1	1.00	1.57	0.278
Pain at week 1	2.06	1.86	0.497
POSAS at week 13			
Observer overall	2.69	2.58	0.799
Patient overall	3.38	3.46	0.408

## Data Availability

The data presented in this study are available on reasonable request from the corresponding author. The data are not publicly available due to data privacy restrictions.
